# Health checkup profile before age 65 and long-term care needs: a retrospective analysis of the Fukuoka-City Information Platform for Community-based Integrated Care

**DOI:** 10.1265/ehpm.26-00086

**Published:** 2026-10-02

**Authors:** Takanori Honda, Hiroko Furuhashi, Emi Oishi, Yoshihiko Furuta, Yasumi Kimura, Toshiharu Ninomiya

**Affiliations:** 1 Department of Epidemiology and Public Health, Graduate School of Medical Sciences, Kyushu University, Fukuoka, Japan; 2 Department of Medical Informatics, Faculty of Medical Sciences, Kyushu University, Fukuoka, Japan; 3 Department of Health and Nutrition, Faculty of Health Sciences, Hiroshima Shudo University, Hiroshima, Japan

**Keywords:** Long-term care needs, Japanese, Retrospective study, Administrative database

## Abstract

**Background:**

The association between health status before age 65 and the occurrence of long-term care needs has not been extensively studied. We aimed to examine potential associated factors based on profiles from Japan’s Specific Health Checkup program that are associated with the occurrence of long-term care needs in later life.

**Method:**

A total of 13,379 residents aged 57–64 years without long-term care needs who underwent Japan’s Specific Health Checkup in fiscal year 2012 were followed until March 2021. The occurrence of long-term care needs was defined as new certification for long-term care level 1 or higher under Japan’s universal long-term care insurance system. Associations between health checkup profiles and the occurrence of long-term care needs were estimated using a Cox proportional hazards model.

**Results:**

During the median 8-year follow-up, 406 participants were newly certified as requiring long-term care. Older age, female sex, underweight, hypertension, diabetes, proteinuria, history of chronic disease (cerebrovascular disease, heart disease, and renal failure), current smoking, no regular exercise, self-reported slow walking speed, skipping breakfast, and eating before bedtime were significantly associated with higher risk of the need for long-term care in age- and sex-adjusted analyses.

**Conclusions:**

Using a large-scale database of participants aged less than 65 years, this study found the significant associations between several health- and lifestyle-related characteristics and the subsequent need for long-term care. Our findings suggest that information collected through health checkups in midlife may be useful for characterizing individuals who may be more likely to require long-term care later in life.

**Trial registration:**

NA

**Supplementary information:**

The online version contains supplementary material available at https://doi.org/10.1265/ehpm.26-00086.

## Introduction

As the global population ages, the social and economic burden associated with long-term care needs among older adults has become a critical global issue [1]. Japan is among the most rapidly aging countries worldwide and is therefore expected to face an extremely high burden as the number of individuals requiring long-term care due to functional limitation increases [2]. Therefore, a fundamental strategy for the early detection and prevention of long-term care needs is essential.

Japan has a universal, mandatory social insurance for long-term care, known as the long-term care insurance system [3]. Under this system, all residents aged 65 or older are eligible for long-term care services if they are recognized as having functional decline, regardless of cause, income level, or availability of caregivers [4]. The certification criteria for long-term care needs are standardized nationwide and serve as indicators of functional disability. Consequently, previous research has examined risk factors for long-term care needs based on these certifications within Japan’s universal long-term care insurance system [5]. However, due to the nature of the system, most studies to date have focused only on older adults aged 65 years or older at baseline. As a result, most candidate associated factors have been identified from data collected after individuals reached age 65.

Investigating associated factors before age 65 may provide new insights into early identification and monitoring of individuals who may be more likely to require long-term care [6, 7]. To address this gap, we explored early associated factors for the occurrence of long-term care needs by linking data from Japan’s universal long-term care insurance system with other medical and health information. In particular, for insurers such as companies and local governments, utilizing existing health checkup information can reduce the costs associated with introducing new geriatric tests and enable them to effectively assess the long-term care insurance costs for their insured individuals.

The Fukuoka-City Information Platform for Community-based Integrated Care Database links data from Japan’s Specific Health Checkups (*Tokutei Kenshin*) with long-term care certification information at the individual level, with linkage based on the Basic Resident Registry [8]. This database enables us to examine the relationship between individual health checkup profiles collected before age 65 and subsequent occurrence of long-term care certification. Therefore, using this database, the present study aimed to examine associations between health checkup profiles from the Specific Health Checkup before age 65 and the subsequent occurrence of long-term care needs in later life.

## Methods

### Design and population

This study was a retrospective cohort study using data from the Fukuoka-City Information Platform for Community-based Integrated Care Database. Details of this database have been described elsewhere [8]. At the time of the analysis, the data from April 2012 through March 2021 were available. Health insurance beneficiaries are required to undergo annual health checkups, so we set the baseline period as fiscal year 2012 (i.e., April 2012 to March 2013). Of the 746,867 people insured by the National Health Insurance (NHI) program in April 2012, 158,582 people aged 57 to 64 years (i.e., those expected to reach 65 years during the follow-up period) were included. After excluding 1,991 who had already been certified as requiring long-term care by the end of fiscal year 2012, 156,591 individuals were followed up for long-term care needs certification. Of those, 3,294 whose status regarding long-term care needs certification during follow-up was missing (i.e. withdrawal from the long-term care insurance due to relocation by April 2013 etc.) and 139,918 who did not receive a Specific Health Checkup during fiscal 2012 were excluded, leaving 13,379 individuals for analysis.

This study was conducted in accordance with the principles of the Declaration of Helsinki and the Ethical Guidelines for Medical and Biological Research Involving Human Subjects in Japan [9]. This study was approved by the Kyushu University Institutional Review Board of Clinical Research (approval number 2021–254). The institutional review board determined that informed consent was not required since this is a secondary use of anonymized information obtained from administrative services.

### Occurrence of long-term care needs

The occurrence of long-term care needs was defined as new certification for long-term care level 1 or higher [10] under Japan’s universal long-term care insurance system during the follow-up period from April 2013 to March 2021, as identified in data provided by the municipal government office. Certification of long-term care needs was based on a nationally standardized procedure for assessing physical and mental functional capacity [11]. Specifically, the need for long-term care is determined by combining a uniform computer-assisted standard needs-assessment system together with a physician’s assessment, followed by a review by a panel of experts [3]. For those identified as needing long-term care, care needs were categorized into seven levels: support levels 1–2 and care levels 1–5.

### Candidate factors

We selected candidate factors potentially associated with the occurrence of long-term care needs associated from among the items collected in the Specific Health Checkup. In Japan, residents aged 40–74 years receive the Specific Health Checkup each year under the universal health insurance system [12], and the items included in the Specific Health Checkup are standardized nationwide. As mentioned above, we used data from the Specific Health Checkup conducted in fiscal year 2012 as the baseline. Considering the available data from Specific Health Checkups and the existing evidence on risk factors for new certification of long-term care needs [13–16], we selected the following candidate factors: age, sex, obesity, underweight, hypertension, diabetes, dyslipidemia, elevated γ-glutamyl transpeptidase (γ-GTP) levels, proteinuria, glycosuria, history of cerebrovascular disease, history of heart disease, history of renal failure or dialysis, anemia, current smoking, frequency of alcohol consumption, weight gain of ≥10 kg since age 20, engaging in at least 30 min of exercise twice per week, self-reported walking speed compared with peers, skipping breakfast ≥3 times per week, self-reported eating speed, eating within 2 h before bedtime ≥3 times per week, snacking habits, and whether sleep provided adequate rest.

Details of the measurement standards for each item have been described elsewhere [8]. Body mass index (BMI) levels of ≥25 kg/m^2^ and <18.5 kg/m^2^ were classified as obesity and underweight, respectively, according to widely used cut-offs for Japanese population [17]. Hypertension was defined as systolic blood pressure ≥140 mmHg, diastolic blood pressure ≥90 mmHg, or use of antihypertensive medication. Diabetes was defined as fasting blood glucose ≥126 mg/dL, hemoglobin A1c levels of ≥6.5%, or use of glucose-lowering medication. Dyslipidemia was defined as serum triglycerides ≥150 mg/dL, high-density lipoprotein cholesterol <40 mg/dL, low-density lipoprotein cholesterol ≥140 mg/dL, or use of lipid-lowering medication. A high γ-GTP level was defined as ≥50 U/L. History of cerebrovascular disease, history of heart disease, history of renal failure or dialysis, and anemia were determined by self-report [18]. Items on lifestyle habits were also collected via self-reported questionnaire [18]. Smoking status was categorized as current or not. Alcohol consumption was categorized as having heavy drinking defined as >2 *go* (in *sake* equivalents) of alcoholic beverages per drinking day or not. The following items were assessed using a yes/no format: weight gain of 10 kg or more since age 20, exercising at least per week for at least 30 min each time, walking speed faster than peers, skipping breakfast 3 or more times per week, having dinner within 2 h before bedtime at least 3 times a week, and sleep (adequate rest from sleep). Snacking between meals was categorized as every day or not. Eating speed was categorized as fast or normal/slow, according to the standard guide for Specific Health Checkup [18]. As mentioned above, questionnaire items are nationally standardized, and all items are selected for inclusion in the Specific Health Checkup program following reviews by a specialized subcommittee of the Ministry of Health, Labour and Welfare, taking their validity and clinical significance into account [18].

### Statistical analysis

The Cox proportional hazards model was used to estimate hazard ratios and 95% confidence intervals for the occurrence of long-term care needs associated with each candidate factor. Follow-up was conducted on a monthly basis and censored at the earlier of the occurrence of long-term care needs or the loss of insured status (due to death or moving out of the city). The models were adjusted for age and sex at the baseline examination. As a supplementary exploratory analysis, we also fitted a multivariable-adjusted model that included all candidate factors. The estimates from this model were interpreted as conditional associations and not as causal effects or independent etiologic contributions. We also performed a sensitivity analysis in which new certification of requiring long-term support level 1 or higher (i.e., any long-term certification regardless of level) was used as the outcome. As an additional analysis, we performed a Cox proportional hazards model to estimate hazard ratios for a composite outcome of long-term care needs and all-cause death. This takes into account a competing risk of death. In other words, death was considered as censoring to estimate the risk of receiving a long-term care certification in the main analysis; however, there may be cases in which individuals died due to a sudden deterioration in health and, as a result, missed the opportunity to receive certification. Therefore, we used a composite outcome of long-term care certification or death to examine whether deaths before certification materially affected the observed associations. All analyses were performed using SAS version 9.4 (SAS Institute, Cary, NC), and two-sided p-values of <0.05 were considered statistically significant.

## Results

Figure 1 presents the study flowchart. The final sample included 13,379 individuals. Table 1 summarizes the baseline characteristics, including physical examination and lifestyle habits, of participants in 2012. The mean age was 61.4 years, and 36% of participants were male. The prevalence of hypertension, diabetes, and dyslipidemia at the 2012 health checkup was 39.6%, 9.1%, and 56.7%, respectively. Among lifestyle habits, common responses included no exercise for at least 30 min twice per week, and slower walking speed than peers. Other frequently reported lifestyle factors included weight gain of ≥10 kg since age 20, fast eating speed, daily alcohol consumption, and insufficient rest from sleep. S-Table 1 in Additional file 1 shows the comparison between individuals for whom health checkup data was available (i.e. those included in the study) and those for whom it was not (i.e. those excluded from the study). The individuals included in the study were older (aged 60–64) and had a higher proportion of women compared to those excluded.

**Fig. 1:**
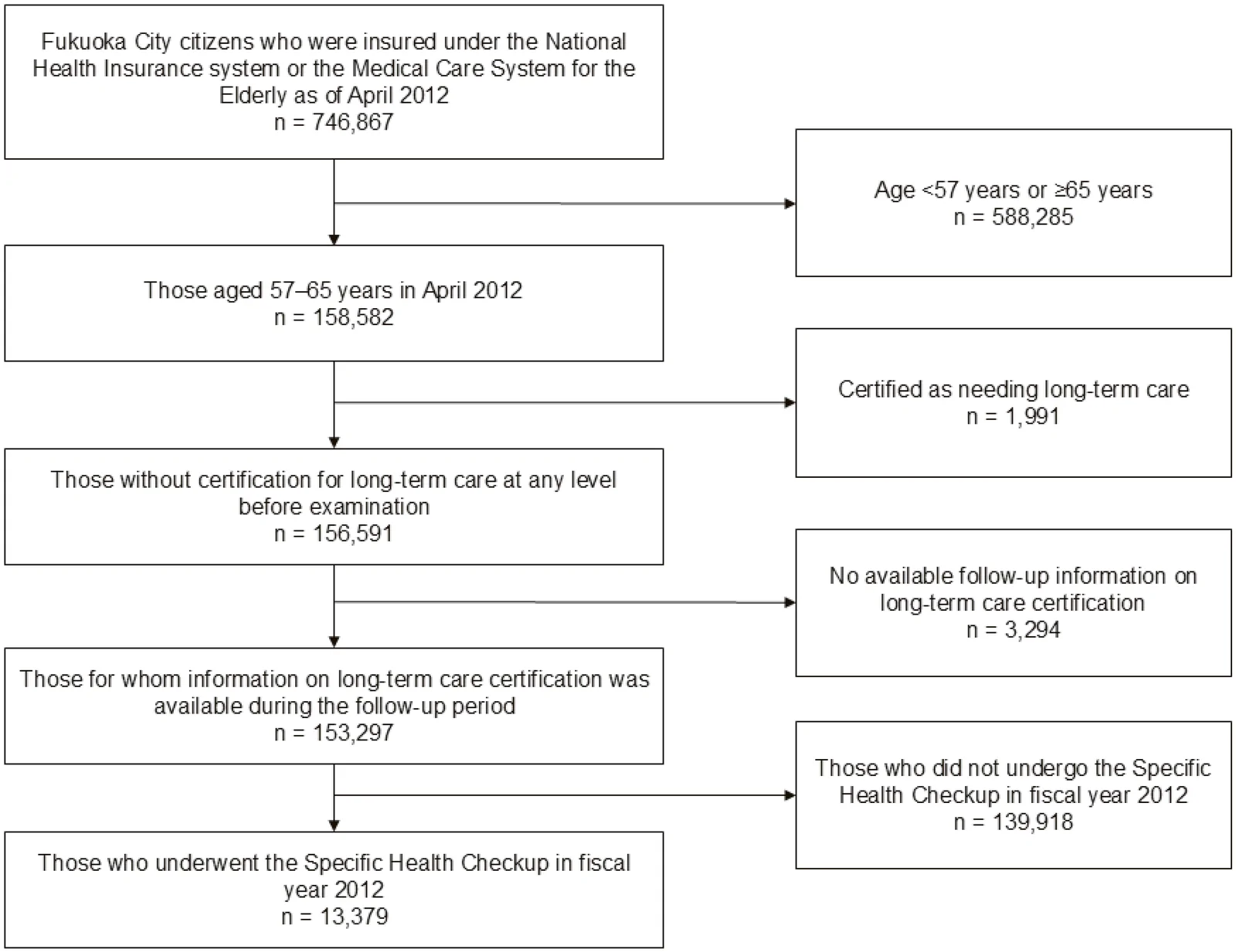
Study flowchart

**Table 1:** Participant characteristics at the baseline examination in 2012

	**Number of** **participants** **analyzed**	**N with** **missing** **values**	**Mean (standard** **deviation) or** **frequency**
Age, years	13,379	0	61.4 (2.1)
Men, %	13,379	0	35.9
BMI, kg/m^2^	13,379	0	22.5 (3.3)
Hypertension, %	13,379	0	39.6
Systolic blood pressure, mmHg	13,379	0	125.6 (16.9)
Diastolic blood pressure, mmHg	13,379	0	75.5 (10.7)
Diabetes, %	13,379	0	9.1
Fasting blood glucose, mg/dL, median (IQR)	11,605	1774	91 (85–99)
Hemoglobin A1c, %	13,377	2	5.7 (0.7)
Dyslipidemia, %	13,379	0	56.7
Serum triglycerides, mg/dL, median (IQR)	13,379	0	97 (71–136)
Serum high-density lipoprotein cholesterol, mg/dL	13,379	0	66.8 (17.6)
Serum low-density lipoprotein cholesterol, mg/dL	13,379	0	127.8 (30.8)
Serum γ-GTP, U/L, median (IQR)	13,379	0	23 (17–38)
Proteinuria, yes (+ to 3+), %	13,376	3	4.9
Urine glucose, yes (+ to 3+), %	13,376	3	2.3
History of cerebrovascular disease, %	13,379	0	2.6
History of heart disease, %	13,379	0	4.9
Kidney failure and/or dialysis, %	13,379	0	0.2
Anemia, %	13,379	0	13.0
Current smoking, %	13,379	0	15.2
Heavy drinking, 2 *go* or more per drinking day, %	13,366	13	8.9
Weight gain of 10 kg or more since age 20, %	13,355	24	33.2
Exercise at least twice per week for at least 30 min each time, %	13,344	35	61.0
Walking speed faster than peers, %	13,287	92	42.5
Skip breakfast 3 or more times per week, %	13,344	35	12.4
Eating speed, fast, %	13,371	8	27.7
Dinner within 2 h before bedtime at least 3 times a week, %	13,372	7	16.7
Snacking between meals, %	13,365	14	14.0
Adequate rest from sleep, %	13,305	74	25.5

BMI, body mass index; γ-GTP, γ-glutamyl transpeptidase; IQR, interquartile range

HbA1c is presented as a National Glycohemoglobin Standardization Program (NGSP) equivalent value (%).

During the median follow-up period of 8 years, 406 people were newly certified as requiring long-term care need level 1 or higher, and 147 were certified as requiring support level 1 or 2, resulting in 553 people being certified as any levels of requiring long-term care. As of March 2021, 404 persons had died. S-Table 2 in Additional file 1 summarizes the baseline characteristics in participants with and without any certification for long-term care.

Table 2 demonstrated age- and sex-adjusted hazard ratios for the occurrence of long-term care need for the candidate associated factors. We observed significant associations of several checkup profiles including older age, female sex, having underweight, hypertension, diabetes, proteinuria, and a history of chronic diseases (cerebrovascular disease, heart disease, and renal failure), current smoking, no regular exercise, slow walking speed, skipping breakfast, and eating before bedtime with the higher risk of requiring long-term care. As a supplementary exploratory analysis, we performed multivariable-adjusted analysis for the occurrence of long-term care needs among 13,109 participants after excluding participants with missing values for candidate factors (n = 270). As shown in S-Table 3, older age, female sex, underweight, diabetes, history of cerebrovascular disease, history of renal failure or dialysis, current smoking, and slower walking speed showed statistically significant conditional associations with the subsequent need for long-term care.

**Table 2:** Age- and sex-adjusted hazard ratios for the occurrence of long-term care needs

	**Hazard ratio** **(95% confidence intervals)**	**P value**
Age, per 1-year increase	1.21 (1.15–1.28)	<0.001
Sex, women vs. men	1.98 (1.63–2.40)	<0.001
Obesity, vs. normal	1.13 (0.90–1.42)	0.31
Underweight, vs. normal	1.88 (1.39–2.53)	<0.001
Hypertension, yes vs. no	1.43 (1.17–1.74)	0.00
Diabetes, yes vs. no	2.54 (2.00–3.23)	<0.001
Dyslipidemia, yes vs. no	1.04 (0.85–1.26)	0.73
High γ-GTP level, ≥50 U/L vs. <50 U/L	1.19 (0.93–1.53)	0.17
Proteinuria, positive (+ to 3+) vs. negative (− to ±)	1.56 (1.10–2.21)	0.01
History of cerebrovascular disease, yes vs. no	2.80 (1.94–4.02)	<0.001
History of heart disease, yes vs. no	1.79 (1.29–2.48)	<0.001
History of renal failure/dialysis, yes vs. no	6.68 (2.76–16.17)	<0.001
Anemia, yes vs. no	1.14 (0.84–1.56)	0.40
Current smoking, yes vs. no	1.47 (1.15–1.87)	0.002
Heavy drinking, 2 *go* and more per drinking day, yes vs. no	1.28 (0.95–1.73)	0.10
Weight gain of 10 kg or more since age 20, yes vs. no	1.14 (0.93–1.39)	0.22
Exercise at least twice per week for at least 30 min each time, no vs. yes	1.33 (1.09–1.63)	0.01
Walking speed faster than peers, no vs. yes	2.02 (1.66–2.47)	<0.001
Skip breakfast 3 or more times per week, yes vs. no	1.44 (1.11–1.87)	0.01
Eating speed, fast vs. normal/slow	0.89 (0.71–1.11)	0.30
Dinner within 2 h before bedtime at least 3 times a week, yes vs. no	1.38 (1.09–1.75)	0.01
Snacking between meals, yes vs. no	0.94 (0.70–1.26)	0.67
Adequate rest from sleep, no vs. yes	1.05 (0.84–1.32)	0.68

γ-GTP, γ-glutamyl transpeptidase.

Variables were adjusted for age and sex: the hazard ratio estimate for age was adjusted for sex, and that for sex was adjusted for age, respectively.

A sensitivity analysis in which new certification of requiring long-term support level 1 or higher (i.e. any levels of certification) was used as the outcome did not materially change the observed results compared with the original outcome definition, although the association between obesity and weight gain and the outcome became statistically significant (see S-Table 4 in Additional file 1). In an additional analysis that considered a composite outcome of long-term care needs and all-cause death, we observed generally similar associations, with all factors identified as associated in the initial analysis remaining statistically significant (see S-Tables 5 in Additional file 1). We further found significant associations of high γ-GTP level, heavy drinking, and obtaining adequate rest from sleep with the risk for the composite outcome.

## Discussion

In this retrospective cohort study of community-dwelling Japanese residents aged 57–64 years at baseline, we found that various factors including physical conditions and lifestyle behaviors from Specific Health Checkups, including older age, female sex, having underweight, hypertension, diabetes, proteinuria, and a history of chronic diseases, current smoking, no regular exercise, slow walking speed, skipping breakfast, and eating before bedtime, were associated with an elevated risk of the need for long-term care. Similar associations were observed in analyses based on the outcome indicating a milder level of care needs, and in analyses based on a composite outcome of long-term care and death. These findings provide information on characteristics in midlife that are associated with the subsequent need for long-term care in midlife. While most previous studies exploring associated factors for long-term care needs in Japan were conducted in populations aged over 65 years [5], our study provides unique insights by using health checkup profile data collected prior to this age. In particular, history of chronic diseases and healthy lifestyle in midlife were associated with the subsequent need for long-term care in later life. In addition, given that many individuals requiring long-term care are likely already receiving medical care for pre-existing conditions [19], information from regular health checkups in midlife may be useful for characterizing individuals who may be more likely to require long-term care and for informing subsequent monitoring. In this study, we were able to obtain these findings using a database that integrates distinct data sources: health checkups and long-term care. Consolidating information across administrative databases could help population-based monitoring for long-term care needs by local governments.

A scoping review showed that most previous studies exploring risk factors for long-term care needs in Japan were conducted in populations aged >65 years [5], and very few studies have used exposures or physical conditions measured prior to age 65. Of the 91 studies on risk factors for long-term care certification identified in the scoping review by Takahashi et al. [5], only four nested case-controlled studies had a baseline measurement at <65 years of age. Our findings, which identified chronic diseases (e.g., diabetes and cerebrovascular disease) and lifestyle factors (e.g., smoking and walking speed) as significant predictors, are largely consistent with previous reports targeting populations aged 65 years or older. Moreover, since the study participants were aged 57–64 years at baseline and were followed for a median of 8 years, long-term care certifications observed in this study likely occurred before 75 years of age. Therefore, the present study addresses relatively early-onset long-term care needs, which represent an important public health issue. A major methodological advantage of the present study is the focus on the pre-elderly stage (aged 57–64 years). In addition, previous exploratory studies of associated factors often focused on items that reflect consequences and signs of functional disability such as items on activities of daily living in the Kihon Checklist [20, 21]. In contrast, the factors included in the present study are included in nation-wide general health checkups for adults; thus, our findings suggest the potential utility of information collected through existing health checkup systems for characterizing individuals who may be more likely to require long-term care.

According to the recent Comprehensive Survey of Living Conditions by the Japanese Ministry of Health, Labour and Welfare, dementia and cerebrovascular disease are the two most common diseases associated with the need for long-term care in Japan [19]. Hypertension, diabetes, and smoking are well-known common risk factors for dementia and cerebrovascular disease [22], which may partly explain their associations with the subsequent need for long-term care observed in the present study. Furthermore, epidemiological studies have shown that the long-term presence of these factors from middle age is associated with elevated risk of dementia, regional brain atrophy, and sarcopenia in later life [23–26]: In the present study, a lack of exercise and certain eating habits were also associated with the higher risk of long-term care needs. A previous study has demonstrated an association of healthy lifestyle habits, including physical activity and dietary behavior, with the risk for long-term care [16]. These findings also support the results of this study. In the supplementary mutually adjusted analysis, some of these associations were attenuated after adjustment for physical conditions, chronic diseases, and lifestyles. However, this attenuation should be interpreted cautiously because the included characteristics may be interrelated, and their causal relationships could not be determined in the present study. Despite these limitations, routinely collected information on these factors may be potentially useful for characterizing individuals who may be more likely to require long-term care in primary care settings.

We observed that underweight and slow walking speed were significant predictors of the occurrence of long-term care needs in later life. This is consistent with previous research findings on physical frailty and functional disability [27]. Our findings suggest that early signs of frailty may be detectable even in the “pre-elderly” stage, and can be captured by simple health checkup items. Unlike many studies that focus on obesity as a primary risk factor for chronic diseases in midlife [28], our findings underscore the importance of focusing on underweight in health checkup and primary care settings. Indeed, there is consistent evidence that underweight is associated with functional disability and dementia [29, 30]. On the other hand, previous findings on obesity and the risk for long-term care are not consistent. Interestingly, we observed that obesity was a significant risk factor only in the sensitivity analysis in which the outcome was defined as being certified for support level 1 or above. According to the 2022 Comprehensive Survey of Living Conditions, dementia was the leading cause of certification for care-need levels, while joint disease was the major cause of the certification for support levels [19]. Given that obesity is a well-known risk factor for osteoarthritis [31], which is a common cause of joint disease, our findings may reflect this difference in the underlying factors contributing to long-term care needs. Our results suggest that public health campaigns should emphasize not only preventing obesity in middle age but also underweight as a precursor of the risk of requiring long-term care.

In this study, proteinuria and renal failure and dialysis were strongly associated with the occurrence of long-term care needs. This is in line with a previous study reporting that the urine albumin-to-creatinine ratio strongly predicted the incidence of long-term care needs in community dwelling older adults [32]. As mentioned above, diabetes is known to be associated with physical and cognitive decline [23–26]. Hypertension has also been shown to be an early risk factor for dementia and changes in brain structure [33, 34]. Since renal failure and dialysis are end-stage manifestations of kidney dysfunction and typically develop after prolonged diabetes and hypertension, our findings may reflect kidney dysfunction due to accumulated vascular and metabolic impairment. Given our findings, a simple urine qualitative test in health checkups may help characterize individuals who may be more likely to require long-term care.

The strengths of this study include its large-scale dataset and the nationally standardized definition of long-term care certification, which ensures high comparability with previous findings in Japanese populations. Furthermore, a key feature of the present study is that it examines the association between factors from Specific Health Checkups assessed at 57–64 years of age and subsequent long-term care certification after 65 years of age over a median follow-up period of 8 years. This age range is also clinically and publicly relevant because developing long-term care needs before 75 years of age represents a relatively early onset of care dependency. However, limitations should also be noted. First, there is a possibility of selection bias at multiple stages. The study focused exclusively on NHI beneficiaries, which include the self-employed, primary industry workers, non-regular employees, and the unemployed. The NHI beneficiaries have different characteristics from beneficiaries of the Employees’ Health Insurance, which covers the majority of Japanese middle-aged population [35]. Consequently, there are limitations to the external validity of the current study. Furthermore, this analysis was limited to individuals who underwent the Specific Health Checkup, which may threaten the internal validity of the results. In fact, compared to those for whom no health checkup data was available (and who were therefore excluded from the study), the participants for whom data was available tended to be older and more likely to be female. This suggests the presence of a certain degree of healthy participant bias. Second, the retrospective cohort design utilizing a database may have introduced bias, as information on exposure variables may not have been blinded when determining long-term care needs certification. Although the certification process is standardized nationwide, there may have been variation in the decision for the certification because it involves opinions from clinicians and reviewers’ judgment. However, since similar associations were observed even when considering death as a composite outcome, our findings may not have been distorted by such bias. Third, due to the limited study period, the study could only cover a maximum of seven years before age 65. Future studies with a longer study period are needed to fully capture the long-term association of health profiles at an earlier stage in middle age. Fourth, several important factors for long-term care needs, such as socioeconomic status, depression, and sensory impairments, were not examined in the present study. Future research should aim to incorporate these factors to provide a more comprehensive understanding of midlife associated factors for long-term care needs. Finally, long-term care certification in Japan is based on a standardized nationwide assessment system and can be considered a valid administrative indicator of care dependency. However, because it is an administrative outcome under the long-term care insurance system, it may not be identical to a direct clinical measure of physical or cognitive disability. Nevertheless, as all outcomes were defined among individuals aged ≥65 years, for whom certification is determined by care needs regardless of the underlying disease, the influence of disease-specific eligibility criteria is likely limited.

## Conclusions

This study, using a large-scale database of individuals before age 65, identified several key associated factors for the occurrence of long-term care needs in later life. Our findings suggest that information collected through health checkups in midlife may be useful for characterizing individuals who may be more likely to require long-term care later in life. Furthermore, linking information from distinct databases such as those for health checkups and long-term care certification is expected to facilitate efficient monitoring by local governments and the national government.

## Abbreviations

γ-GTP: γ-glutamyl transpeptidase
BMI: Body mass index

## Supplementary information

**Additional file 1:** **S-Table 1.** Comparison of sex and age group between individuals who participated in the Specific Health Checkups (i.e. included in the study population) and those who did not (i.e. excluded). **S-Table 2.** Participant characteristics at the baseline examination in 2012 among individuals with and without long-term care needs during follow-up period. **S-Table 3.** Multivariable-adjusted hazard ratios for the occurrence of long-term care needs. **S-Table 4.** Age and sex-adjusted hazard ratios for new certification of requiring long-term support level 1 or higher. **S-Table 5.** Age- and sex-adjusted hazard ratios for the composite outcome of long-term care needs and all-cause death.

## Declarations

### Availability of data and materials

The datasets generated and analyzed during the current study are not publicly available due to contractual restrictions that do not allow the external publication of individual-level data, but summary information is available from the corresponding author on reasonable request.
