# Supplementary material for: Health checkup profile before age 65 and long-term care needs: a retrospective analysis of the Fukuoka-City Information Platform for Community-based Integrated Care
Source: Environ Health Prev Med. 2026 Oct 2;31:67. doi: 10.1265/ehpm.26-00086 (PMC13649616; doi:10.1265/ehpm.26-00086)
Supplement: Supplementary file 1 [file ehpm-31-067-s001.docx]

| S-Table 1. Comparison of sex and age group between individuals who participated in the Specific Health Checkups (i.e. included in the study population) and those who did not (i.e. excluded). | | | |
| --- | --- | --- | --- |
|  |  | Included | Excluded |
|  |  | (N = 13,379) | (N = 139,918) |
| Men |  |  |  |
|  | 55-59 years | 964 (7%) | 24,049 (17%) |
|  | 60-64 years | 3,843 (29%) | 44,680 (32%) |
| Women |  |  |  |
|  | 55-59 years | 1,816 (14%) | 24,844 (18%) |
|  | 60-64 years | 6,756 (50%) | 46,345 (33%) |

| S-Table 2. Participant characteristics at the baseline examination in 2012 among individuals with and without long-term care needs during follow-up period | | |
| --- | --- | --- |
|  | Mean (standard deviation) or frequency | |
|  | Without long-term care need (N = 12,973) | With long-term care need (N = 406) |
| Age, years | 61.4 (2.1) | 62.2 (1.9) |
| Men, % | 35.4 | 52.0 |
| BMI，kg/m^2^ | 22.5 (3.3) | 22.8 (3.9) |
| Hypertension, % | 39.2 | 52.2 |
| Systolic blood pressure, mmHg | 125.5 (16.8) | 128.7 (19.0) |
| Diastolic blood pressure, mmHg | 75.4 (10.7) | 77.5 (11.8) |
| Diabetes, % | 8.7 | 22.4 |
| Fasting blood glucose, mg/dl | 91 (85-99) | 95 (87-109) |
| Hemoglobin A1c, % | 5.7 (0.7) | 6.0 (1.1) |
| Dyslipidemia, % | 56.7 | 57.6 |
| Serum triglycerides, mg/dL | 97 (71-136) | 103 (77-145) |
| Serum high-density lipoprotein cholesterol, mg/dL | 66.9 (17.5) | 63.3 (18.3) |
| Serum low-density lipoprotein cholesterol，mg/dL | 127.9 (30.6) | 123.1 (34.4) |
| Serum γ-GTP，U/L, median (IQR) | 23 (17-38) | 27 (18-46) |
| Proteinuria, yes (+ to 3+), % | 4.8 | 8.6 |
| Urine glucose, yes (+ to 3+), % | 2.1 | 8.1 |
| History of cerebrovascular disease, % | 2.4 | 7.9 |
| History of heart disease, % | 4.8 | 9.9 |
| Kidney failure and/or dialysis, % | 0.2 | 1.2 |
| Anemia, % | 13.0 | 11.6 |
| Current smoking, % | 15.0 | 23.2 |
| Heavy drinking, 2 go or more per drinking day, % | 8.7 | 13.5 |
| Weight gain of 10 kg or more since age 20, % | 33.0 | 39.2 |
| Exercise at least twice per week for at least 30 min each time, % | 60.9 | 64.4 |
| Walking speed faster than peers, % | 42.0 | 58.9 |
| Skip breakfast 3 or more times per week, % | 12.2 | 16.5 |
| Eating speed, fast, % | 27.8 | 26.7 |
| Dinner within 2 h before bedtime at least 3 times a week, % | 16.5 | 22.9 |
| Eating between meals, % | 14.1 | 12.3 |
| Adequate rest from sleep, % | 25.6 | 24.5 |
| BMI, body mass index; γ-GTP, γ-glutamyl transpeptidase; IQR, interquartile range  HbA1c is presented as a National Glycohemoglobin Standardization Program (NGSP) equivalent value (%). | | |

| S-Table 3. Multivariable-adjusted hazard ratios for the occurrence of long-term care needs | | |
| --- | --- | --- |
|  | Hazard ratio (95% confidence intervals) | P value |
| Age, per 1-year increase | 1.22 (1.16-1.29) | <0.001 |
| Sex, women vs. men | 1.55 (1.23-1.96) | <0.001 |
| Obesity, vs. normal | 0.92 (0.69-1.22) | 0.55 |
| Underweight, vs. normal | 2.15 (1.57-2.95) | <0.001 |
| Hypertension, yes vs. no | 1.20 (0.97-1.49) | 0.10 |
| Diabetes, yes vs. no | 2.37 (1.84-3.07) | <0.001 |
| Dyslipidemia, yes vs. no | 0.96 (0.78-1.18) | 0.71 |
| High γ-GTP level, ≥50 U/L vs. <50 U/L | 0.96 (0.73-1.25) | 0.74 |
| Proteinuria, positive (+ to 3+) vs. negative (- to ±) | 1.17 (0.82-1.68) | 0.38 |
| History of cerebrovascular disease, yes vs. no | 2.19 (1.50-3.19) | <0.001 |
| History of heart disease, yes vs. no | 1.32 (0.93-1.88) | 0.12 |
| History of renal failure/dialysis, yes vs. no | 3.79 (1.51-9.51) | 0.005 |
| Anemia, yes vs. no | 1.04 (0.75-1.43) | 0.83 |
| Current smoking, yes vs. no | 1.30 (1.01-1.67) | 0.045 |
| Heavy drinking, 2 *go* and more per drinking day, yes vs. no | 1.18 (0.86-1.62) | 0.31 |
| Weight gain of 10 kg or more since age 20, yes vs. no | 1.08 (0.84-1.38) | 0.56 |
| Exercise at least twice per week for at least 30 min each time, no vs. yes | 1.16 (0.94-1.44) | 0.17 |
| Walking speed faster than peers, no vs. yes | 1.82 (1.49-2.24) | <0.001 |
| Skip breakfast 3 or more times per week, yes vs. no | 1.25 (0.95-1.65) | 0.12 |
| Eating speed, fast vs. normal/slow | 0.91 (0.73-1.15) | 0.44 |
| Dinner within 2 h before bedtime at least 3 times a week, yes vs. no | 1.22 (0.94-1.57) | 0.13 |
| Snacking between meals, yes vs. no | 0.85 (0.62-1.16) | 0.30 |
| Adequate rest from sleep, no vs. yes | 0.91 (0.72-1.15) | 0.44 |
| Participants with any missing data on potential associated factors were excluded (n = 270).  Variables were mutually adjusted.  γ-GTP, γ-glutamyl transpeptidase. | | |

| S-Table 4. Age and sex-adjusted hazard ratios for new certification of requiring long-term support level 1 or higher | | |
| --- | --- | --- |
|  | Hazard ratio (95% confidence intervals) | P value |
| Age, per 1-year increase | 1.23 (1.18-1.29) | <0.001 |
| Sex, women vs. men | 1.16 (0.98-1.37) | 0.09 |
| Obesity, vs. normal | 1.54 (1.27-1.86) | <0.001 |
| Underweight, vs. normal | 1.33 (1.01-1.74) | 0.04 |
| Hypertension, yes vs. no | 1.60 (1.35-1.89) | <0.001 |
| Diabetes, yes vs. no | 2.18 (1.74-2.72) | <0.001 |
| Dyslipidemia, yes vs. no | 1.09 (0.92-1.30) | 0.30 |
| High γ-GTP level, | 1.04 (0.82-1.32) | 0.75 |
| Proteinuria, positive (+ to 3+) vs. negative (- to ±) | 1.42 (1.02-1.97) | 0.04 |
| History of cerebrovascular disease, yes vs. no | 3.76 (2.81-5.02) | <0.001 |
| History of heart disease, yes vs. no | 1.86 (1.39-2.49) | <0.001 |
| History of renal failure/dialysis, yes vs. no | 7.85 (3.50-17.61) | <0.001 |
| Anemia, yes vs. no | 1.01 (0.78-1.31) | 0.96 |
| Current smoking, yes vs. no | 1.46 (1.17-1.82) | 0.008 |
| Heavy drinking, 2 *go* and more, yes vs. no | 1.31 (0.99-1.74) | 0.06 |
| Weight gain of 10 kg or more since age 20, yes vs. no | 1.34 (1.12-1.59) | 0.001 |
| Exercise at least twice a week for at least 30 min each time, no vs. yes | 1.32 (1.10-1.57) | 0.002 |
| Walking speed faster than peers, no vs. yes | 2.46 (2.07-2.93) | <0.001 |
| Skip breakfast 3 or more times per week, yes vs. no | 1.40 (1.11-1.77) | 0.005 |
| Eating speed, fast vs. normal/slow | 0.99 (0.82-1.19) | 0.89 |
| Dinner within 2 h before bedtime at least 3 times a week, yes vs. no | 1.25 (1.01-1.55) | 0.04 |
| Snacking between meals, yes vs. no | 1.08 (0.85-1.37) | 0.53 |
| Adequate rest from sleep, no vs. yes | 1.12 (0.93-1.35) | 0.25 |
| γ-GTP, γ-glutamyl transpeptidase. | | |

| S-Table 5. Age- and sex-adjusted hazard ratios for the composite outcome of long-term care needs and all-cause death | | |
| --- | --- | --- |
|  | Hazard ratio (95% confidence intervals) | P value |
| Age, per 1-year increase | 1.12 (1.08-1.17) | <0.001 |
| Sex, women vs. men | 2.42 (2.08-2.82) | <0.001 |
| Obesity, vs. normal | 1.04 (0.87-1.24) | 0.66 |
| Underweight, vs. normal | 1.94 (1.53-2.45) | <0.001 |
| Hypertension, yes vs. no | 1.32 (1.13-1.54) | <0.001 |
| Diabetes, yes vs. no | 2.23 (1.85-2.69) | <0.001 |
| Dyslipidemia, yes vs. no | 0.98 (0.84-1.14) | 0.79 |
| High γ-GTP level, | 1.27 (1.05-1.52) | 0.01 |
| Proteinuria, positive (+ to 3+) vs. negative (- to ±) | 1.58 (1.22-2.06) | 0.001 |
| History of cerebrovascular disease, yes vs. no | 2.10 (1.54-2.87) | <0.001 |
| History of heart disease, yes vs. no | 1.52 (1.16-1.98) | 0.002 |
| History of renal failure/dialysis, yes vs. no | 6.54 (3.39-12.65) | <0.001 |
| Anemia, yes vs. no | 1.22 (0.96-1.55) | 0.11 |
| Current smoking, yes vs. no | 1.69 (1.41-2.01) | <0.001 |
| Heavy drinking, 2 *go* and more, yes vs. no | 1.38 (1.11-1.71) | 0.004 |
| Weight gain of 10 kg or more since age 20, yes vs. no | 1.09 (0.93-1.27) | 0.28 |
| Exercise at least twice a week for at least 30 min each time, no vs. yes | 1.36 (1.16-1.60) | <0.001 |
| Walking speed faster than peers, no vs. yes | 1.71 (1.47-1.99) | <0.001 |
| Skip breakfast 3 or more times per week, yes vs. no | 1.27 (1.03-1.57) | 0.02 |
| Eating speed, fast vs. normal/slow | 0.86 (0.72-1.01) | 0.07 |
| Dinner within 2 h before bedtime at least 3 times a week, yes vs. no | 1.38 (1.15-1.65) | <0.001 |
| Snacking between meals, yes vs. no | 0.95 (0.76-1.19) | 0.65 |
| Adequate rest from sleep, no vs. yes | 1.31 (1.11-1.55) | 0.001 |
| γ-GTP, γ-glutamyl transpeptidase. | | |
